# Severe Cases of Tick-Borne Encephalitis in Northeastern Poland

**DOI:** 10.3390/pathogens14010007

**Published:** 2024-12-27

**Authors:** Justyna Adamczuk, Magdalena Chlabicz, Natalia Koda, Maciej Kondrusik, Joanna Zajkowska, Piotr Czupryna, Anna Moniuszko-Malinowska

**Affiliations:** 1 Department of Infectious Diseases and Neuroinfections, Medical University of Bialystok, 15-540 Bialystok, Poland; mkondrusik@poczta.onet.pl (M.K.); joanna.zajkowksa@umb.edu.pl (J.Z.); avalon-5@wp.pl (P.C.); annamoniuszko@op.pl (A.M.-M.); 2 Students’ Scientific Club at the Department of Infectious Diseases and Neuroinfections, Medical University of Bialystok, 15-540 Bialystok, Poland; chlabicz.m@gmail.com (M.C.); nataliakoda2@gmail.com (N.K.)

**Keywords:** tick-borne encephalitis, viral infection, meningoencephalitis, severe case

## Abstract

Tick-borne encephalitis (TBE) is the most prevalent viral infection of the central nervous system (CNS) in Poland. The disease is characterized by the presence of two stages. The first phase, called the viremic stage, presents with flu-like symptoms, while the second stage of TBE is characterized by damage to the nervous system and may follow a severe and dramatic course. The aim of this paper is to increase the awareness of the potential sequelae after TBE. In this study, we report cases of severe TBE in 36-year-old and 57-year-old female patients. The outcome of TBE varies from patient to patient, but there are some factors that can help to predict the severity of TBE infection. The risk factors presented in these patients were as follows: the monophasic course of TBE, high pleocytosis in cerebrospinal fluid (CSF) and older age. Both of our patients were not vaccinated despite the World Health Organization’s (WHO’s) recommendations. Both patients had no history of travel outside their region of residence prior to the onset of illness. The few risk factors shown in our patients and the serious sequelae of the disease may indicate the need to test patients for possible gene mutations.

## 1. Introduction

Tick-borne encephalitis (TBE) is the most prevalent viral infection of the central nervous system in Poland [1]. It is transmitted by Ixodidae tick bites and caused by the *Orthoflavivirus* encephalitidis from the *Orthoflavivirus* genus [2]. The prevalence of ticks infected with tick-borne encephalitis virus (TBEV) in endemic areas in Europe usually varies from 0.5% to 5% [3]. Clinical courses and outcomes vary by the subtype of TBEV. The genetic analysis shows the existence of three TBEV subtypes: the European, Siberian, and Far-Eastern subtype. The disease caused by the European subtype has a milder course and better outcome than the diseases caused by the Siberian and Far-Eastern subtypes. TBE cases occur most commonly in the period from April to November, in correlation with the highest tick activity [4]. Infection is largely associated with outdoor activity in wooded areas, such as picking berries, collecting mushrooms or working on farms [3]. However, it is possible to be exposed to the TBEV by consuming unpasteurized dairy products from livestock that have been infected with the virus [5]. The incidence of the disease has been increasing over the past few decades in almost all endemic European and Asian countries [4].

In 2020, 24 countries in Europe reported 3734 cases of confirmed TBE, with a notification rate of 0.9 per 100,000 residents. A noticeable increase can be seen compared with the rate of 0.7 in 2019, and the stable rate of 0.6 from 2016 to 2018. Whereas, in Poland in 2020, the number of confirmed cases was 114, with a notification rate of 0.3 per 100,00 residents. The notification rate in Poland decreased, compared to previous years: 0.6 for 2016, 0.5 for 2017 and 2019, and 0.4 for 2018, respectively [6]. On the other hand, in 2022 and 2023, the number of cases in Poland increased. In 2022, it was 446, with an incidence rate of 1.18, whereas in 2023, the number of cases was 659, with an incidence rate of 1.75 [7].

The course of the disease can be severe, but it can be successfully prevented by vaccination. The number of individuals vaccinated against TBE in the year 2023 in Poland reached 102,147 [8]. In Poland, preventive vaccination is recommended for people living in areas with an increased prevalence of TBE [9]. However, not all of them are vaccinated. Therefore, the aim of this paper is to increase the awareness of the potential sequelae after TBE by the presentation of 2 cases with severe courses.

In both cases, TBE was diagnosed according to the case definition (symptoms of CNS inflammation and TBE specific IgM, IgG antibodies in blood, and TBE specific IgM antibodies in CSF) [10]. Anti-TBE IgM and IgG antibodies were detected using Euroimmun anti-TBE Virus ELISA according to manufacturer’s instructions.

## 2. Case Report I

A 36-year-old female patient with no past history of chronic diseases and reporting a tick bite two weeks before hospitalization was referred to the Department of Infectious Diseases and Neuroinfections in September 2022 with suspected meningitis. Two days before admission, the patient experienced vomiting, weakness, headache, dizziness, and speech disorders. Initially, the patient was diagnosed in the Emergency Department, where a head computed tomography (CT) scan revealed no abnormalities. On admission to the clinic, the patient’s general condition was fair; the patient was conscious, in logical contact, and had a fever up to 39 °C. A physical examination revealed the paralysis of the VII nerve on the left side, paresis of the left upper limb, complete neck stiffness, and positive Kernig’s signs. The patient was not vaccinated against tick-borne encephalitis.

In the Emergency Department, a lumbar puncture was performed, and cerebrospinal fluid with an increased protein concentration (88 mg/dL), pleocytosis (512 cells/μL) and lymphocytes (9%) was obtained. The detailed data can be found in Table 1 and Table 2. During hospitalization, the right upper limb became paralyzed, with the range of mobility limited to the fingers. Periosteal reflexes from the upper limbs were abolished, and the patient was unable to raise her head. In the following hours, the patient felt weakness in the left lower limb, but the muscle strength in the said limb was preserved. However, this limb was subsequently paralyzed; moreover, the paralysis of the masseter muscles and trismus also occurred.

The treatment included drugs reducing the intracranial pressure (mannitol), steroids, antipyretics, antibiotic therapy (ceftriaxone), and intravenous hydration. Despite the treatment, the fever persisted. On the next day of hospitalization, the patient’s condition worsened, with a disturbance to her consciousness and respiratory failure. In a critical general condition, on passive oxygen therapy, the patient was transferred to the intensive care unit (ICU) in order to continue treatment.

Upon admission to the ICU, the patient was in a very serious condition, conscious, with flaccid quadriplegia and inadequate breathing. The patient was intubated, and mechanical ventilation was started. In the following days of hospitalization, the patient’s condition remained persistently serious, with her eyeballs directed upwards and strabismus. In the following days, the patient regained consciousness, followed recommendations to close her eyes, moved her tongue, and exhibited persistent flaccid quadriplegia. On command, the patient was able to perform slight finger movements of the lower limbs.

In the initial period of hospitalization in the ICU, due to circulatory system instability, pressor amine infusion was used. Diuresis was stimulated with a loop diuretic (furosemide). Three cycles of plasmapheresis were performed, followed by two additional cycles. Pathogenic microorganisms (*Klebsiella pneumoniae* MBL NDM, *Pseudomonas aeruginosa*) were cultured in blood, urine, and bronchial tree aspirate. Broad-spectrum antibiotic therapy was administered (meropenem, ertapenem, vancomycin, piperacillin, tazobactam).

After stabilizing the general condition, the patient remained on passive oxygen therapy with a tracheostomy tube, without sedation, conscious, in maintained non-verbal contact, with persistent flaccid four-limb paralysis and weakened swallowing reflex, and was therefore fed by a percutaneous endoscopic gastrostomy (PEG) feeding tube. The patient was then transferred to the Rehabilitation Department for further treatment.

After being discharged from the Rehabilitation Department, the patient’s condition remained serious. After one year, the patient was recumbent, breathing through a tracheostomy, due to the periodic exacerbation of dyspnoea with gasometric disturbances (hypoxemia and hypercapnia), requiring periodic invasive mechanical ventilation at home.

## 3. Case Report II

A 57-year-old female patient with a past history of depression, multilevel discopathy, and two surgeries of the L/S segment of the spine, was referred to the Department of Infectious Diseases and Neuroinfections in July 2022 due to headaches, chills, nausea, vomiting, and dizziness for the past 3 days. Upon admission to the hospital, her fever went up to 40 °C. The patient was possibly bitten by a tick one week before the onset of the symptoms. The patient had not received a vaccination against TBE. On admission, the patient was feeling weak and reported both headaches and nausea. A physical examination revealed neck stiffness (2–3 cm), a positive Romberg test, and fine-wave tremors of the lips and tongue. There were no obvious neurological deficits or pathological reflexes.

Initial laboratory tests showed hyponatremia. A CSF examination revealed inflammatory changes within the lymph fluid, pleocytosis (335 cells/μL), lymphocytes (65%) and protein (64 mg/dL). Serological tests of blood and CSF for TBEV were positive. The detailed data can be found in Table 3 and Table 4.

During the initial period of hospitalization, the general condition of the patient deteriorated. Verbal contact became difficult, the patient felt drowsy and weakened, features of paresis of the left lower limb appeared and a fever up to 39.0 °C occured. The patient’s condition became severe in the upcoming hours. The patient was confused, in limited contact, with impaired coughing and swallowing reflex. She required temporary high-flow oxygen therapy.

An angio-CT scan of the thorax revealed the retention of a small amount of mucus in the tracheal lumen and the prolonged retention of thick mucus in the lumen of the segmental bronchi of inferior lobes of both lungs. Extensive areas of atelectasis (from obstruction of the bronchial lumen) in the dorsal and supra dorsal portions of inferior lobes of both lungs were observed in thoracic CT imaging. The patient was consulted by a pulmonologist. A bronchoscopy was performed, and the retained bronchial secretion (mucous) in the lower lobes was removed. During the following days of hospitalization, an improvement in the patient’s clinical condition was observed. The return of cough and swallowing reflexes was observed. An anti-decubitus treatment and rehabilitation with a physiotherapist was applied.

The patient was bedbound with the preserved limited mobility of her upper limbs P < L, paralysis of both lower limbs P < L, but in logic contact with 15 GCS points, and she was transferred to the Rehabilitation Department of the Regional Hospital in Bialystok.

The patient was evaluated in the outpatient clinic for a scheduled follow-up appointment 10 months after the previous hospitalization. The patient now moves around using a wheelchair. The flaccid paresis of her left lower limb persists and muscular atrophy is still present.

## 4. Discussion

The vaccination coverage against tick-borne encephalitis (TBE) in Poland remains low, with reported rates of only 11%. This contrasts sharply with significantly higher rates observed in other European countries, such as Austria (81%) and Latvia (62%), highlighting a substantial gap in preventive measures against TBE in Polan [11]. TBE caused by the European TBEV subtype is a disease with a mostly bi-phasic course [12]. The initial incubation period lasts between 2 and 28 days, averagely lasting about 7 days. The first phase, called the viremic stage, presents with flu-like symptoms such as mild fever, headache, tiredness, aching back and limbs and nausea. Then comes the period of no clinical symptoms [13]. The second phase of TBE is characterized by a strong response from CNS. A spectrum of clinical manifestations, including high fever, headache, vomiting, convulsions, paresis of the respiratory muscles, paralysis, or even coma, may occur. In severe cases, manifesting as meningoencephalitis with multifocal symptoms or meningoencephalomyelitis, this disease may become deadly after only a week of clinical manifestation [13,14]. Around 75% of European TBE infections remain sub-clinical [15]. TBE manifests as meningitis in about 50% of cases, as meningoencephalitis in about 40%, and as meningoencephalomyelitis in about 10% [16].

The outcome of TBE varies from patient to patient, but some factors may be identified as potentially helpful in predicting the severity of TBE infection, such as immunodeficiency, viral strain, and disease phenotype [14]. Additionally, high levels of pleocytosis and the monophasic course of the disease are considered risk factors for more severe courses of the disease [17].

Furthermore, one of the predictors of the severity of TBE seems to be the presence of detectable serum matrix metalloproteinase 9 (MMP-9) [18]. Patients with meningoencephalitis seem to have significantly higher single nucleotide polymorphism in the MMP-9 gene, rs 17576, than patients with meningitis [19]. MMP-9 was also found in CSF as a predictor of CSF TBEV IgG positivity and corresponding with increased serum IL-6 concentrations which was associated with worse outcomes, such as death [20].

In both described patients, the course of the disease was monophasic and both presented with meningoencephalomyelitis.

Additionally, the long-term morbidity in TBE patients is correlated with the male gender and an older age. Although both of our patients were female, the second patient was 57 years old, which might also be considered a risk factor. As previously stated, in a cohort study by Lenhard T. et al., older patients suffered significantly and more frequently from meningoencephaloradiculitis (62 years) and meningoencephalitis (57 years), than in meningitis (46 years), as well as male patients whom more frequently had meningoencephaloradiculitis (81.2%) and meningoencephalitis (71%), than meningitis (43.3%) [21]. This cohort study also distinguished pre-existing diabetes mellitus as a possible risk factor for meningoencephaloradiculitis.

Nygren T.M. et al. conducted a prospective cohort study, between 2018 and 2020, with a sample of 581 participants with confirmed cases of TBE. The data were collected using questionaries and hospital discharge summaries [22]. They analyzed acute clinical manifestations of TBE and investigated the risk factors of a severe disease course. They showed that age, hypertension and a monophasic course were associated with higher odds of severe TBE. A prospective observational study, carried out in Slovenia from 2007 to 2012 on 717 patients with TBE by Bogovič P. et al. indicated that patients of an older age, without a previous vaccination against TBE, higher blood leukocyte count, higher serum C-reactive protein levels and a low level of specific TBEV serum IgG antibodies were linked to more severe courses of TBE [23]. A retrospective study was conducted by Radzišauskienė D. et al. to describe the clinical and epidemiological features of TBE in adults. A total of 1040 patients were included in the study in the years 2005–2017. The highest proportion of severe cases, reaching 41.2%, was reported in the 70–79 age group. Each additional 10 years of age increases the risk of severe TBE by 30%. Other predictors of the most severe myelitic form highlighted in this study, in addition to the indicated age above 60, were the presence of CNS disease, bulbar syndrome, high pleocytosis in CSF, and the delayed immune response of TBEV IgG [24].

Both our patients presented with high pleocytosis, which is consistent with other reports [17].

## 5. Conclusions

Despite undergoing rehabilitation for 18 months, both patients exhibited minimal improvement. Such cases require ongoing interdisciplinary medical care involving specialists, such as physical therapists, neurologists and otolaryngologists, among others.

In case I, the risk factors of a severe outcome were as follows: a monophasic course, the clinical form of TBE (meningoencephalomyelitis) and high pleocytosis, while in case II, additionally, age might have influenced the outcome.

Regardless of the circumstances, that there were few risk factors shown in our patients indicated their cases were severe, which may indicate the need to genetically test patients for possible gene mutations, such as single nucleotide polymorphism in the MMP-9 gene. Both of our patients were not vaccinated against TBE, which could have prevented the disease and long-term disability. WHO recommends vaccination as the primary preventative measure against TBE with its excellent safety and efficiency profile [25].

## Figures and Tables

**Table 1:** Results of laboratory tests of serum in the reported Case Report I.

Parameter	Value	Reference Range
WBC (10^3^/μL)	9.72	4.0–10.0
RBC (10^6^/μL)	5.05	4.2–5.9
HGB (g/dL)	15.6	13.5–17.5
HCT (%)	43.7	38.8–50.0
CRP	1.6	<3.0
INR	1.19	0.8–1.2
Sodium (mmol/L)	141	135–145
Potassium (mmol/L)	3.5	3.5–5.1
Fibrinogen (mg/dL)	168	200–400
pH	7.3	7.35–7.45
PCO_2_ (mmHg)	47	35–45
PO_2_ (mmHg)	38.3	75–100
TBE IgG (RU/mL)	8.35	Negative < 16
TBE IgM (RU/mL)	2.26	Negative < 1.1

WBC: white blood cells, RBC: red blood cells, HGB: hemoglobin, HCT: hematocrit, INR: international normalized ratio (standardized metric employed to quantitatively assess the coagulation propensity of blood), CRP: c-reactive protein.

**Table 2:** Results of laboratory test of cerebrospinal fluid in the reported Case Report I.

Laboratory Parameter	Value	Reference Range
Total nucleated cell count (μL)	512	<5
Lymphocytes (%)	9	20–40
Monocytes (%)	3	2–8
Macrophages (%)	1	<1
Neutrophils (%)	87	50–70
Glucose (mg/dL)	80	50–80
Protein (mg/dL)	88	15–45
Nonne-Apelt test	(-)	Negative
Pandy’s test	Not done	Negative
Lactic acid (mmol/L)	3.4	0.5–2.2
TBE IgG (U)	16.58	Negative < 5
TBE IgM (U)	>100	Negative < 1.1

**Table 3:** Results of laboratory tests of serum in the reported Case Report II.

Laboratory Parameter	Value	Reference Range
WBC (10^3^/μL)	10.99	4.0–10.0
RBC (10^6^/μL)	4.35	4.2–5.9
HGB (g/dL)	13.1	13.5–17.5
HCT (%)	37.9	38.8–50.0
CRP	5.63	<3.0
INR	1.23	0.8–1.2
Sodium (mmol/L)	133	135–145
Potassium (mmol/L)	4.71	3.5–5.1
pH	7.502	7.35–7.45
PCO_2_ (mmHg)	31.9	35–45
PO_2_ (mmHg)	42.3	75–100
TBE IgG (RU/mL)	175.35	Negative < 16
TBE IgM (RU/mL)	3.5	Negative < 1.1

PCO_2_: partial pressure of carbon dioxide, PO_2_: partial pressure of oxygen.

**Table 4:** Results of laboratory test of cerebrospinal fluid in the reported Case Report II.

Laboratory Parameter	Value	Reference Range
Total nucleated cell count (μL)	335	<5
Lymphocytes (%)	65	20–40
Monocytes (%)	21	2–8
Neutrophils (%)	14	50–70
Glucose (mg/dL)	63	50–80
Protein (mg/dL)	64	15–45
Nonne-Apelt test	(+)	Negative
Pandy’s test	(+++)	Negative
Lactic acid (mmol/L)	3.4	0.5–2.2
TBE IgG (U)	>100	Negative < 5
TBE IgM (U)	45.27	Negative < 5

## Data Availability

The data presented in this study are available on request from the corresponding author.
